# The Nation and Its Nurses

**Published:** 1917-01-06

**Authors:** 


### THE NATION AND ITS NURSES.

### THEIR ENEMIES' TACTICS ARE IDENTICAL.

The war has made such a call upon our womankind, and especially upon the nurses and those possessing nursing instincts, that they must necessarily have been aroused to take a fairly continuous interest in its progress. The trained nurse has greatly strengthened her position by the devotion and excellence of the services she has rendered in war-time, not only to the warriors who have been entrusted to her care, but to the nation, who recognises that her ministrations and the ministrations of members of the kindred profession of medicine have made everybody her or his debtor. The close association that both have had with war in many lands has naturally made them wishful to follow the progress of the fighting.

Indeed, the Nation and British Nurses at the commencement of 1917 have very much in common. Both have enemies to fight, and it is enlightening to realise that the tactics of those enemies are demonstrably identical in spirit. Germany and the Central Committee for the State Registration of Nurses, London, desired, provoked, and declared the wars which, on the one hand, have involved the Allies, and, on the other, the British Nursing Profession. Demonstrably both wars had their origin in the overmastering desire for supreme power possessed, in each case, by a single individual. The Central Empires rejected all attempts to prevent war made by the Entente, *that is the Allies*. The Central Committee, too, rejected all attempts to secure agreement and unity made by the Honourable Arthur Stanley and the representatives of the Nurse-Training Schools and of the College of Nursing, who are known to be *the disinterested friends of British Nurses*. So both the enemies of the Nation and the enemies of the best interests of British Nurses deliberately insisted upon war. Both the Nation's and the Nurses' enemies after some experience of war have come to realise, that neither of the Individuals who are really responsible for the war, in either instance, will ever be permitted to attain to supreme power, in the one case over the Nation, and in the other over British Nurses. It has taken two and a half years to bring the Nation's enemies to this conviction, but the prime mover and organiser of the Central Committee carried on negotiations for peace, until the assured conviction of failure to attain supreme power forced a change of policy, *i.e.* one of indefinite delay and continuous postponement. The Allies have rejected sham offers of peace on the part of Germany. They denounce these overtures and define them as nothing more than a calculated attempt to influence the future course of the war, and to end it by imposing a German peace. For identical reasons and in self-defence British Nurses are opposed to the Central Committee. The test ballot instituted by *The Nursing Mirror*, as we showed last week, demonstrates through representatives of the great body of trained British Nurses in active work, that, British Nurses resent the enemy's policy of disunion, and reprobate their tactics of delay and postponement. Decades of British Nurses are each day placing their names on the College Register, are uniting in support of the Hon. Arthur Stanley, their Parliamentary leader, and standing shoulder to shoulder in defence of their Rights and Independence. British Nurses have indeed rejected, and are daily in increasing numbers uniting to defeat the attempt, which has been proceeding for twenty-five years, to divide the British Nursing Profession.

The British Nursing Profession is fully alive to these facts, and is also seized with the knowledge that the Central Committee for the State Registration of Nurses, London, is mostly make-believe. The claim that it is fully representative of British Nurses and their interests is nonsense, and this claim will be torn to pieces at the first sitting at which it is examined by Parliament, if the Central Committee and its Bill survive until that stage is reached. Presumably these facts are known to the Council of the British Medical Association, in view of that Council's instructions on October 25 last, to its representatives on the Central Committee, "to join with the other bodies represented on that Committee, in any further attempts which may be made to construct an agreed Bill." In this connection it should be remembered that the representatives on the Central Committee of the Royal British Nurses' Association, and of the Association for Promoting the Registration of Nurses in Scotland, that is the overwhelming majority of Scottish Nurses, have already protested against the action and procedure of the Central Committee.

In view of the continuous inflow of fully-qualified nurses who are flocking day by day to be registered with the College of Nursing, through *The Nursing Mirror*, and directly, and the facts here set forth, is it not well for the representatives of the British Medical Association, in conjunction with those of the Royal British Nurses' Association and the Association for the Promotion of the Registration of Nurses in Scotland, to meet together, seek a conference with Mr. Stanley and go solid for an agreed Bill? This course is well within their powers, and as they are entitled to be regarded as independent, public-spirited persons, genuinely anxious to secure a settlement and promote the best interests of British nursing, such an action on their part would surely be a good one to commence the year 1917. Meanwhile, it cannot be pleasant for the Council of the British Medical Association to be served up in print as a Stalking Horse, behind which the Body, which calls itself the Central Committee, masquerades before the public and neutral opinion, as the Blameless Knight of "Energy, Self-Sacrifice, and Courage," fighting against "Privilege, Prejudice, Bluster, Self-Interest, and Cowardice," save the mark!

